# A new genus of water mites (Acari, Hydrachnidia, Wettinidae) from bromeliad phytotelmata in the Brazilian Atlantic rainforest

**DOI:** 10.3897/zookeys.516.10179

**Published:** 2015-08-06

**Authors:** Vladimir Pešić, Gustavo Cauê de Oliveira Piccoli, Marcel Santos de Araújo, José Marcos Rezende

**Affiliations:** 1 Department of Biology, University of Montenegro, Cetinjski put b.b., 81000 Podgorica, Montenegro; 2 Programa de Pós-Graduação em Biologia Animal, São Paulo State University, São José do Rio Preto, SP, 15054-000, Brazil

**Keywords:** Water mite, new genus, taxonomy, Brazil

## Abstract

Adults of *Bromeliacarus cardoso* **gen. n.**, **sp. n.** are described from phytotelmata of *Quesnelia arvensis* (Vellozo) Mez. (Bromeliaceae) in the subtropical area of the Atlantic rainforest, São Paulo State, Brazil. The new genus *Bromeliacarus* is proposed and diagnosed, based primarily on the autapomorphic presence of 7–9 pairs of acetabula flanking the gonopore. A possible relationship between *Bromeliacarus* and other Wettinidae are discussed.

## Introduction

Bromeliads phytotelmata (i.e. tank bromeliads) are considered biodiversity amplifiers in the environments where they occur due to the specificity of a high number of species in this habitat (Rocha et al. 2000, Gonçalves-Souza et al. 2010). Despite the taxonomy and biology of several bromeliad-dwellers, organisms are poorly understood (Frank and Lounibos 2008) and there are continuous studies increasing descriptions of new species and genera in different invertebrates groups such as dipterans (Epler 2010, Pinho et al. 2013), beetles (Clarkson et al. 2014, Albertoni and Fikáček 2014) and water mites (Pešić et al. 2015, present study). The water mite fauna of bromeliad phytotelmata is insufficiently known (Kitching 2000). Although the first paper appeared in the early part of the 20^th^ century (K. Viets 1939), only a few more studies on water mites from this unusual habitat have been published (see review in Kitching 2000 and references in Pešić et al. 2015). Recently, an extensive sampling effort in bromeliad aquatic fauna for ecological studies in Brazilian subtropical area of the Atlantic rainforest provides material to improve taxonomic knowledge of bromelicolous water mites. The first paper resulting from that expedition dealt with the new species of the genus *Xystonotus* (Pešić et al. 2015). In this paper one new genus of the family Wettinidae is described.

After the revision by Cook et al. (2000), Wettinidae is recognized as separate family, which in addition to the Holarctic nominate genus, includes *Stormaxonella* K.O. Viets, 1962 (South Africa), *Tasmanaxona* Cook, 1986, *Wheenyella* Cook, 1986, and *Wheenyoides* Harvey, 1990 (all from Australia). The new genus described here is the first member of Wettinidae to exhibit a polyacetabulate condition, bearing 7-9 acetabula on each side flanking the gonopore. In other characters, notably in the presence of a large central shield surrounded by a ring of small platelets, the new species is similar to *Stormaxonella* K.O. Viets, 1962, a monotypic genus known only from streams in South Africa (K. O Viets 1962). This character state is apparently apomorphic, and may represent synapomorphy indicating a close relationship between *Bromeliacarus* gen. n. and *Stormaxonella* and members of the family Lethaxonidae which have a dorsal shield similar in structure. Cook et al. (2000) wrote about Lethaxonidae: “We interpret modifications of the first and fourth pair of legs found in members of *Lethaxona* and *Lethaxonella* as synapomorphies indicating common ancestry with Wettinidae.” (p. 435). The same authors mentioned that a more detailed analysis of the relationship between the genera of Lethaxonidae and Wettinidae based upon on comparative larval morphology (only known for *Wettina*) is warranted (Cook et al. 2000, p. 441).

## Material and methods

The sampling site is located in State Park of Ilha do Cardoso, São Paulo State, Brazil. This area is included in Atlantic Rainforest domain and shows most of coastal phytophysiognomies of rocky shores, mangroves and restingas (Bernardi et al. 2005). Mites were collected from phytotelmata of *Quesnelia arvensis* (Vellozo) Mez., a bromeliad species with terrestrial and epiphytic habits densely distributed in the understory of restinga. Each leaf of the bromeliad was carefully dissected and washed, and all detritus and water were collected in white trays. Mites and other fauna were extracted and fixed in 80% alcohol. The holotype will be deposited at the Acari Collection of the Departamento de Zoologia e Botânica (DZSJRP), São Paulo State University, Sao José do Rio Preto, São Paulo, Brazil; paratypes are deposited in the Zoological Collection of the Department of Biology, University of Montenegro, Podgorica.

All measurements are given in µm. The following abbreviations are used: Cx-I = first coxae, dL = dorsal length, H = height, L = length, I-L-6 = Leg 1, sixth segment (tarsus), P-1 = palp, first segment, vL = ventral length, W = width.

## Systematics

### Family Wettinidae Cook, 1956

#### Genus Bromeliacarus

Taxon classificationAnimaliaProstigmataWettinidae

Pešić gen. n.

http://zoobank.org/581654AA-B239-4585-BF32-20FACD7359DC

##### Diagnosis.

**Figure 1.**
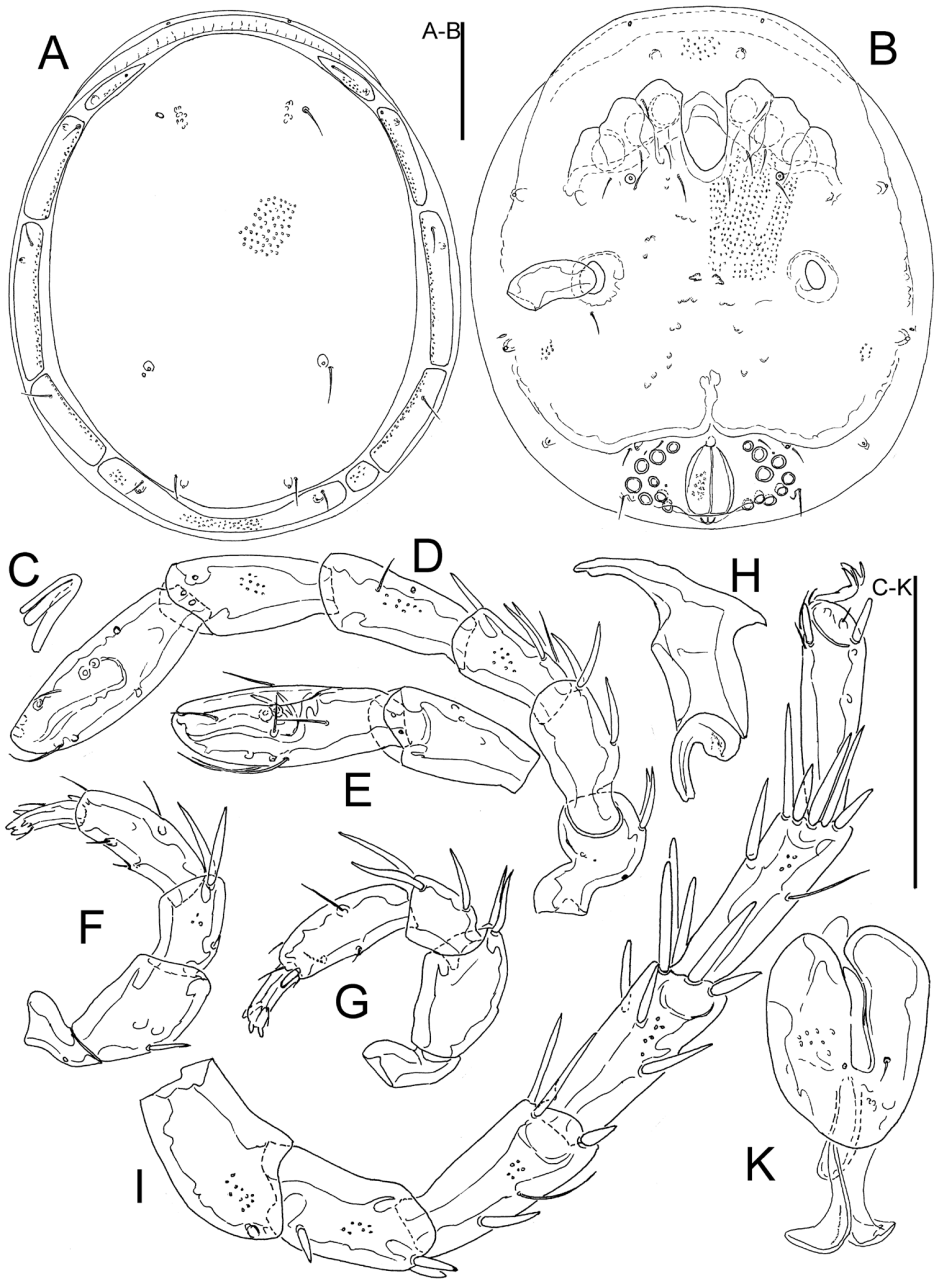
**A–H** *Bromeliacarus cardoso* sp. n., female: **A** idiosoma, dorsal view **B** idiosoma, ventral view **C** claw of first leg **D** I-L **E** I-L-5 and -6 **F** palp, lateral view **G** palp, medial view **H** chelicera **I** IV-L **K** gnathosoma. Scale bars: 100 µm.

**Figure 2.**
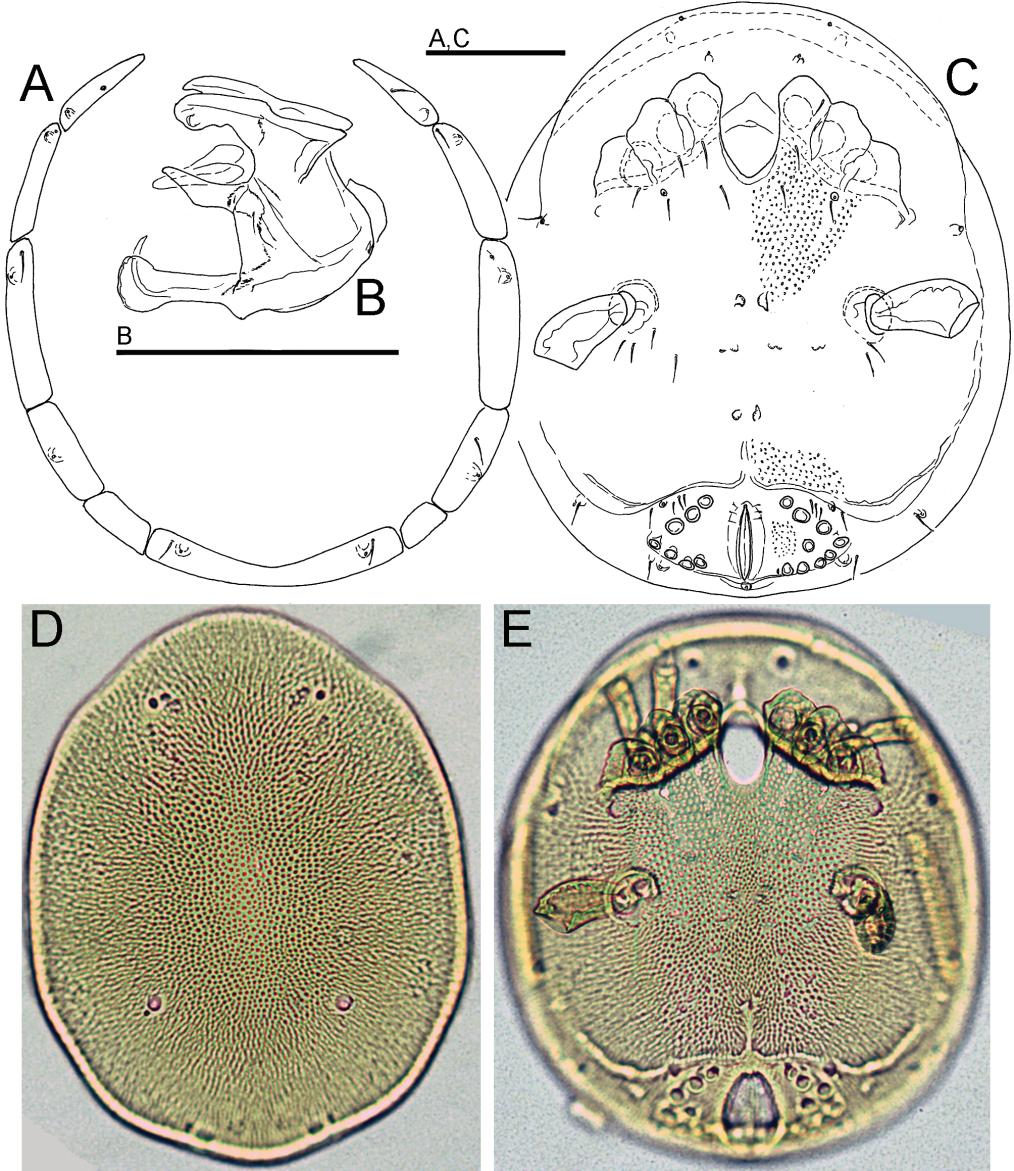
**A–E** *Bromeliacarus cardoso* sp. n. (**A, D–E** female **B–C** male) **A–C** line drawing **D–E** photographs: **A** ring of platelets surrounding dorsal plate **B** gnathosoma **C, E** idiosoma, ventral view **D** dorsal plate. Scale bars: 100 µm.

Characters of the family Wettinidae (see Cook et al. 2000): dorsum with a large central shield bearing two pairs of glandularia and a pair of postocularia (Fig. 2D), flanked by ring composed of 6 pairs of platelets (Figs 1A, 2A), with 1^th^, 2^nd^, 3^rd^, 4^th^, and 6^th^ pairs bearing glandularia; 6^th^ pair of platelet fused to each other, occasionally on one side 5^th^ platelet fused with 4^th^ platelet. Venter with coxal plates and genital field fused into a ventral shield (Figs 1B, 2C, 2E); suture lines indicating posterior edges of anterior three coxal plates weakly indicated but directed posteriorly; IV-L insertion laterally, well separated from each other and without projections; coxoglandularia 1 in posterior edges of Cx-II; Cx-IV without glandularia; coxoglandularia 2 between Cx-IV and genital field. Legs: I-L stocky, I-L-6 with a long and deep claw socket extending over more than half the dorsal segment surface (Fig. 1E), claw large, ventral clawlet apically rounded, slightly longer than main claw (Fig. 1C); legs I-IV without swimming setae. Genital field with 7-9 pairs of acetabula; acetabular plates fused with ventral shield in both sexes (Figs 1B, 2C); suture lines between genital field and ventral shield obliterated; excretory pore incorporated into ventral shield. Gnathosoma with relatively long apodemes (Fig. 1K); palp slender, P-4 bearing two short ventral setae inserting in the centre and a peg-like mediodistal seta (Fig. 1F–G).

##### Type species.

*Bromeliacarus cardoso* sp. n.

##### Etymology.

Named for its occurrence in bromeliad phytotelmata, and the Latin *acarus* meaning “*mite*”.

##### Remarks.

Adults of *Bromeliacarus* gen. n. share with those of all Wettinidae the apomorphic characteristic modifications of first leg (short and stocky with tarsal claw sockets exceptionally large and claws large with ventral clawlet slightly longer than main claw) and fourth leg (trochanter being long and massive, tarsal claw sockets reduced) and the posterior orientation of the suture lines between coxal plates. This new species is autapomorphic in having 7-9 pairs of genital acetabula flanking the gonopore. Other members of Wettinidae differ in exhibiting the plesiotypic character state of small number of genital acetabula (i.e., *Stormaxonella* K.O. Viets, 1962 with four pairs of acetabula, all other genera with three pairs of acetabula but one species of *Wettina* (*Wettina octopora* Cook) with four pairs. Due to the similar structure of dorsal shield, the new genus appears to be related to *Stormaxonella* K.O. Viets, 1962. This character state is apomorphic and may indicate that both genera belong to a monophyletic group within Wettinidae. However in light of striking difference in genital field and palp (*Stomoxanella scutulata* is autapomorphic in P-4 bearing one thick, spatulate seta medially in proximal third of segment) it would appear that divergence from a common ancestor have occurred early during wettinid evolution. Cook et al. (2000; 437) claim that “*the occurence of different clades on widely separated land masses in both the Northern and Southern Hemispheres suggests that members of this family were distributed throughout Pangea before it broke apart during the Jurassic*”.

#### Bromeliacarus cardoso

Taxon classificationAnimaliaProstigmataWettinidae

Pešić sp. n.

http://zoobank.org/8A11B848-9412-4A5A-9C0A-53A970719CE1

Figs 1 , 2

##### Type series.

Holotype female, dissected and slide mounted in Hoyer’s fluid, Brazil, São Paulo, Cananéia, 25°04'16"S, 47°55'23"W, in *Quesnelia arvensis* (Vellozo) Mez. (Bromeliaceae), v.2013 col. Gustavo Cauê de Oliveira Piccoli. Paratype: three females (two of them damaged, palps and legs lacking), one male (damaged, palps and legs lacking), same data as holotype, two females (both damaged) and one male dissected and slide mounted in Hoyer’s fluid.

##### Diagnosis.

As given for genus.

##### Description.

Character states as given in generic diagnosis.

##### Measurements.

Female (holotype, in parentheses some measurements of paratype): Idiosoma (ventral view: Fig. 1B) L/W 434/375 (441-456/367-400). Dorsal shield (Figs 1A, 2D) L/W 363/308 (398-409/309-322), ratio 1.18 (1.27–1.29); gnathosomal bay L 69 (78); distance between IV-leg insertions 172 (173); gonopore L/W 69/39 (63/40), distance between most lateral pairs of Ac 146 (151). Palp (Figs 1F-G): total L 177; L/H, L/H ratio: P-1, 25/14, 1.8; P-2, 44/28, 1.6; P-3, 32/20, 1.6; P-4, 48/15, 3.1; P-5, 28/8, 3.4; gnathosoma vL 72, with apodemes 105; chelicera total L 86. Legs: dL of I-L (Fig. 1D): 41, 51, 40, 52, 51, 74; I-L-6 H 26, I-L-6 dL/H ratio 2.8; dL of II-L-2-6: 59, 44, 55, 64, 72; dL of III-L-2-6: 56, 45, 59, 72, 72; dL of IV-L (Fig. 1I): 75, 56, 66, 69, 71, 74.

Male: Idiosoma (ventral view: Fig. 1C) L/W 434/375. Dorsal shield L/W 384/306, ratio 1.26; gnathosomal bay L 77; distance between IV-leg insertions 167; gonopore L/W 54/6, distance between most lateral pairs of Ac 148.

##### Etymology.

Named after the locality (State Park of Ilha do Cardoso, São Paulo, Brazil) where the new species was detected.

##### Variability.

The number of acetabula flanking the gonopore varies from 7 to 9 on each side. We found three different combinations Ac numbers flanking (right+left) the gonopore: 7+9 (one male), 8+8 (one female) and 8+9 (two females).

##### Distribution.

Brazil; only known from the type locality.

##### Habitat and biology.

Members of *Bromeliacarus cardoso* sp. n., are unusual about their habitats, because they appear to live only in the water-filled leaf axils of the bromeliads, where they walk attached to submerged detritus in bromeliads tank or free swimming in water column. Additional collecting effort is clearly needed in order to understand life history as well as habitat preferences of this species. However, as already mentioned by Albertoni and Fikáček (2014), the usual method used for searching for fauna inside bromeliads, i.e. dismantling the leaves one by one and washing the content in a tray, may not to be effective enough for very small species.

## Supplementary Material

XML Treatment for Bromeliacarus

XML Treatment for Bromeliacarus cardoso

## References

[B1] Albertoni FF Fikáček M (2014) A new bromeliad-inhabiting species of *Omicrus* Sharp from South Brazil (Coleoptera, Hydrophylidae, Sphaeridiinae). Spixiana 37: 111–122.

[B2] Cook DR Smith IM Harvey MS (2000) Assessment of lateral compression of the idiosoma in adult water mites as a taxonomic character and reclassification of *Frontipodopsis* Walter, *Wettina* Piersig and some other basal Hygrobatoidea (Acari: Hydrachnidia). Invertebrate Taxonomy 14: 433–448. doi: 10.1071/IT99014

[B3] Clarkson B Albertoni FF Fikáček M (2014) Taxonomy and biology of the bromeliad-inhabiting genus *Lachnodacnum* (Coleoptera: Hydrophilidae: Sphaeridiinae). Acta Entomologica Musei Nationalis Pragae 54: 157–194.

[B4] Bernardi JVE Landim PBM Barreto CL Monteiro RC (2005) Spatial study of the vegetation gradient from Cardoso Island State Park, SP, Brazil. Holos Environment 5: 1–22.

[B5] Epler JH (2010) *Phytotelmatocladius*, a new genus from bromeliads in Florida and Brazil (Diptera: Chironomidae: Orthocladiinae). Proceedings of the XV International Symposium on Chironomidae, 285–293.

[B6] Frank JH Lounibos LP (2008) Insects and allies associated with bromeliads: a review. Terrestrial Arthropod Reviews 1: 125–153. doi: 10.1163/187498308X414742 20209047 10.1163/187498308X414742 PMC2832612

[B7] Gonçalves-Souza T Brescovit AD Rossa-Feres DC Romero GQ (2010) Bromeliads as biodiversity amplifiers and habitat segregation of spider communities in a Neotropical rainforest. Journal of Arachnology 38: 270–279. doi: 10.1636/P09-58.1

[B8] Kitching RL (2000) Food webs and container habitats: the natural history and ecology of phytotelmata. Cambridge University Press, Cambridge. doi: 10.1017/CBO9780511542107

[B9] Pinho LC Mendes HF Andersen T Marcondes CB (2013) Bromelicolous *Polypedilum* Kieffer from South Brazil (Diptera: Chironomidae). Zootaxa 3652: 569–581. doi: 10.11646/zootaxa.3652.5.6 26269856 10.11646/zootaxa.3652.5.6

[B10] Rocha CFD Cogliatti-Carvalho L Almeida DR Freitas AFN (2000) Bromeliads: biodiversity amplifiers. Journal of Bromeliads Society 50: 81–83.

[B11] Viets KO (1962) Neue Gattungen und Arten von Wassermilben aus Südafrika. Zoologischer Anzeiger 168(7–10): 356–388.

[B12] Viets K (1939) Eine neue, die erste Süßwassermilbe (Hydrachnellae, Acari) aus tropischen Pflanzgewässern. Zoologischer Anzeiger 128(3–4): 69–77.

[B13] Pešić V Piccoli GCO Araújo MS Rezende JM Gonçalves AZ (2015) A new species of *Xystonotus* Wolcott, 1900 (Acari, Hydrachnidia, Mideopsidae) from bromeliad phytotelmata in Brazilian Atlantic rainforest. Zootaxa 3981(1): 147–150. doi: 10.11646/zootaxa.3981.1.10 26249986 10.11646/zootaxa.3981.1.10

